# Single-Readout High-Density Memristor Crossbar

**DOI:** 10.1038/srep18863

**Published:** 2016-01-07

**Authors:** M. A. Zidan, H. Omran, R. Naous, A. Sultan, H. A. H. Fahmy, W. D. Lu, K. N. Salama

**Affiliations:** 1 Department of Electrical Engineering & Computer Science, University of Michigan, Ann Arbor, MI 48109, USA; 2 Electrical Engineering Program, King Abdullah University of Science and Technology (KAUST), Thuwal 23955, Saudi Arabia; 3 Electronics and Communications Department, Faculty of Engineering, Cairo University, Giza 12316, Egypt

## Abstract

High-density memristor-crossbar architecture is a very promising technology for future computing systems. The simplicity of the gateless-crossbar structure is both its principal advantage and the source of undesired sneak-paths of current. This parasitic current could consume an enormous amount of energy and ruin the readout process. We introduce new adaptive-threshold readout techniques that utilize the locality and hierarchy properties of the computer-memory system to address the sneak-paths problem. The proposed methods require a single memory access per pixel for an array readout. Besides, the memristive crossbar consumes an order of magnitude less power than state-of-the-art readout techniques.

Current processor and memory technologies face design challenges that are related to the continuous scaling down of the minimum feature size anticipated by Moore’s Law^1^. Moreover, conventional computing architecture is no longer an effective way to meet the demands of modern applications. An exigent need therefore exists to shift to new technologies at both architectural and device levels. Recently, the high-density, memristor-crossbar architecture has attracted attention in this regard. Memristor based resistive RAM is a promising candidate to replace Hard Disk Drive (HDD), DRAM, and flash memories^1^,^2^,^3^,^4^,^5^,^6^,^7^. Moreover, the high-density, memristive-crossbar is also a perfect candidate for neural bio-inspired computing^8^,^9^,^10^,^11^,^12^. Such applications are driven by recent advances in the fabrication of memristive devices^13^,^14^,^15^,^16^,^17^,^18^,^19^,^20^.

The main advantage of a redox memristive array is its very high density^1^,^21^, which entails that each memory cell occupies few nanometers. The array is simply built as a crossbar structure. Such simple assembly is inherently self-aligned and can be fabricated using only one or two lithography masks^13^. While the simplicity of the structure is its principal advantage, it is also the source of its main problem, namely the sneak-paths problem^21^,^22^. While accessing the array, current should flow only through the desired cell. However, nothing in the crossbar prevents the current from sneaking through other cells in the array as shown in Fig. 1a,b. This parasitic current ruins the reading and writing operations, and consumes a considerable amount of power.

The direct solution to the sneak-paths problem is to add a selector (gate) to each memory cell such as: MOS transistors^23^, threshold devices^24^, and complementary memristors^2^,^25^. In general, this comes at the expense of array density and the complexity of the fabrication process (cost per bit)^21^. Hence, the need arises to address the sneak-paths challenge using the typical gateless crossbar structure but with a quality similar to that of the gated arrays. Several techniques have been proposed for handling such an effect in gateless arrays, including multistage readout^5^, multiport readout^3^, unfolded arrays^26^, engineering device nonlinearity^27^, and grounded array^21^. However, these techniques either require extended accessing time, rely on a power-hungry accessing, reduce the density of the array significantly, or are even not a valid solution for practical size arrays^21^.

In this work, we introduce single-stage readout techniques for the high-density gateless resistive arrays. The new method reduces the access time to the crossbar array significantly based upon the sneak-paths correlation analysis and the locality property of memory systems. The new readout adopts a very power efficient mode of access to the crossbar, guided by the study of the sneak-paths power consumption presented in this work. In addition, minimal control and sensing circuitry are required. Altogether, compared to previous work presented in the literature, we offer a faster and more power-efficient readout with a simple sensing mechanism.

## Sneak-Paths Correlation Analysis

**Figure 1:**
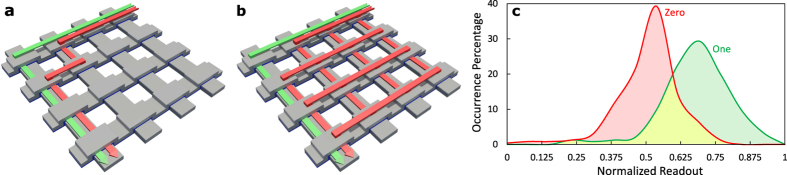
Crossbar with (**a**) a single sneak-path and (**b**) current sneaking throughout the whole array.(**c**) Histogram for the “One” and “Zero” values distributions as a result of the sneak-paths effect for a 256k array filled with NIST RAM images [28].

Sneak-paths impact the performance of a crossbar-based system in two ways. First, a considerable amount of undesirable energy is consumed as current sneaks throughout the array cells. Second, the sneak currents cannot be predicted because they are data dependent. Data stored in a memory array is naturally random^28^, which leads to a random sneak-paths resistance. This is translated into having distributions that represent the “One” and “Zero” values rather than a single value. In addition, the magnitude of the sneak-current is typically higher than the current of the desired memory cell^3^; hence the distributions for the two binary values are highly overlapped, as shown in Fig. 1c. Direct memory readout is therefore not possible; thus, a power efficient sneak-paths immune readout is a necessity for a functional system.

**Figure 2:**
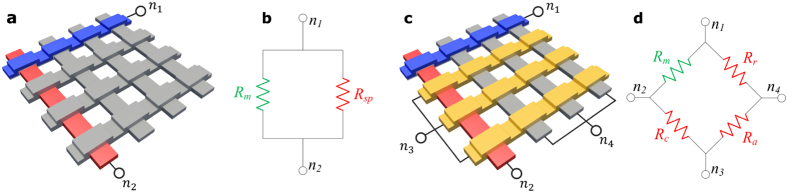
(**a**) Floating terminals accessing mode and (**b**) its equivalent circuit. (**c**) Connected terminals accessing mode and (**d**) its equivalent circuit.

One of the generally utilized properties of the sneak-paths current is its spatial correlation. Knowing the sneak-path noise value at one location of the crossbar helps to estimate the values at other correlated locations. Engineering such properties enables us to propose faster and more power-efficient readout techniques for the resistive crossbar memories. In general, a crossbar can be accessed using two modes: “floating terminals” and “connected terminals”, as shown in Fig. 2. In the first approach, the selected array terminals are kept floating. On the other hand, in the “connected terminals” approach the selected rows and columns are connected to two common nodes. The two extra nodes can be used as access terminals to the array^3^, or to enforce a bias voltage^29^. This allows for better control of the sneak-paths behavior and yields a more usable equivalent circuit. In such case, the sneak-paths are represented by three lambed resistances (‘*R*_*r*_’, ‘*R*_*a*_’ and ‘*R*_*c*_’) as shown in Fig. 2d. Understanding the correlation of these elements over the crossbar facilitates a better handling of the sneak-paths noise. For instance, ‘*R*_*r*_’ is a parallel combination of all the desired row cells apart from the desired one; it is given by, 
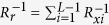
, where ‘*R*_*x*_‘ is the resistance of a one-row cell, and ‘*L*’ is the array length. The row cell resistance can be either ‘
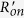
’ or ‘
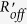
’, which are the ON and OFF resistance of the device under ‘*V*_*n*1_ − *V*_*n*4_’ voltage drop respectively. The row resistance can be rewritten as,


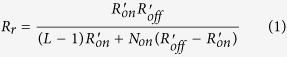


where ‘*N*_*on*_’ is the number of ON cells within the accessed row not counting the accessed cell itself. The remaining two sneak-path components (*R*_*c*_ and *R*_*a*_) have similar expressions. Although, in the case of biasing the unused array terminals the sneak-paths component‘*R*_*a*_’ is shorted out. It should be noted that although the metal line resistances are not included in the equivalent circuit for sake of simplicity, they have been fully considered in the simulations carried out in this work.

**Figure 3:**
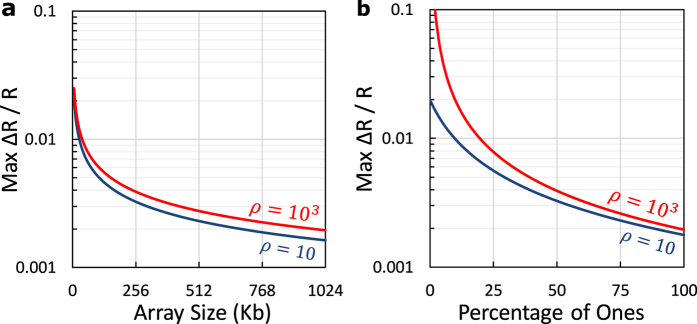
Maximum change in resistance related to its original value versus (**a**) the array size with balanced ones and zeros, and (**b**) percentage of ones for an array of a size of 256 kb. ‘Δ*R*/*R*’ represents both the ‘Δ*R*_*r*_/*R*_*r*_’ and the ‘Δ*R*_*c*_/*R*_*c*_’.

For practical array size, the values of ‘*R*_*r*_’ and ‘*R*_*c*_’ are almost constant over the same row or column respectively. For instance, the sneak-paths row resistances found at two different locations in the same row have all cells in common except the two cells that are swapped because of the accessed locations. For devices with a large OFF/ON ratio, the relative change in the sneak-paths row resistance is given by,


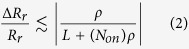


where ‘*ρ*’ is the OFF/ON ratio of the used device. The maximum relative change in the row resistance versus the array size for a balanced number of zeros and ones is plotted in Fig. 3a. The figure shows that as the array size increases the effect of a single bit swap diminishes. The other parameter that affects Δ*R*/*R* is the number of ones (per row or column), as given by (2). Figure 3b shows that the maximum relative change of sneak-paths resistance is still small while the percentage of ones per row/column is swept. Hence, ‘*R*_*r*_’ is almost constant over a given row and ‘*R*_*c*_’ is almost constant over a given column. It should be noted that, given the randomness of the data, ‘*R*_*r*_’ and ‘*R*_*c*_’ are considered two independent random variables.

## Adaptive-Threshold Readout Techniques

The sneak-paths correlation property can be effectively utilized in case of sequential reading for the stored data on a memory array. The good news is that this is the typical memory access scheme in computer systems. Because of the memory-locality property, data is transferred and shared between different memory layers as a block of contiguous bits, rather than in random bits or words^30^. This locality property is of help only if the knowledge gained from reading a single bit can be adopted in reading its neighborhoods. This is true for the “connected terminals” crossbar, where the values of ‘*R*_*r*_’ and ‘*R*_*c*_’ can be safely shared over the same row or column, respectively, as discussed in the previous sections. This is equivalent to defining an adaptive threshold that changes at each new row readout, which can be achieved with the aid of the “connected terminals” crossbar.

**Figure 4:**
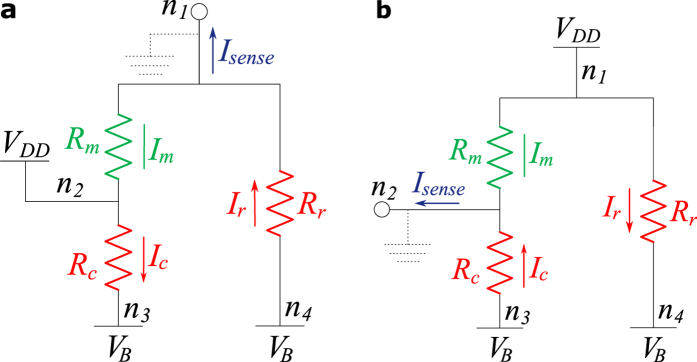
Equivalent circuit for the “connected terminals” accessing mode when the row and column terminals are forced to the same voltage and the sense circuit is connected to either the desired (**a**) row or (**b**) column.

**Figure 5:**
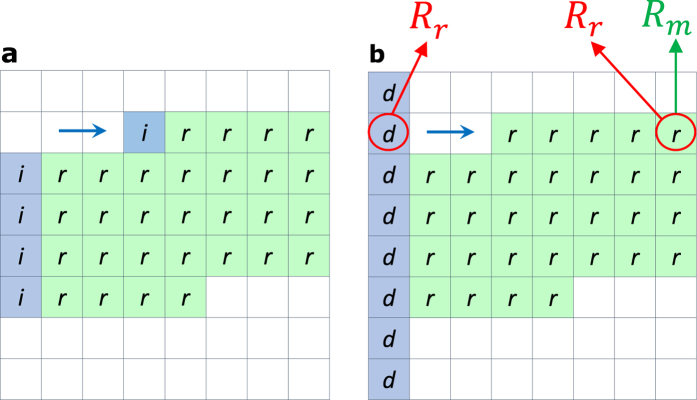
(**a**) Array accessing sequence, where the initial bit per row/column is accessed ‘*n*’ times, while the rest of the bits in the same row/column are accessed once. (**b**) The accessing sequence in case of using predefined “dummy bits”, where all of the bits of the array are accessed in a single stage fashion. d: dummy bit, i: initial bit, and r: regular bit.

The generic “connected terminals” circuit model shown in Fig. 2d can be simplified for the case of ‘*V*_*B*_’ terminals bias. Terminals ‘*n*_3_’ and ‘*n*_4_’ are connected to ‘*V*_*B*_’, and terminals ‘*n*_1_’ and ‘*n*_2_’ are connected to ‘*V*_*DD*_’ and virtual ground. This can be done with two different implementations as shown in Fig. 4. Using a virtual ground sensing circuit forces all of the array elements to have a defined voltage drop independent of the data stored in the array. The desired cell experiences a full ‘*V*_*DD*_’ voltage drop, while the sneak-paths components of ‘*R*_*r*_’ and ‘*R*_*c*_’ have a voltage drop of ‘*V*_*DD*_ − *V*_*B*_’. Because of the device saturation nonlinearity, the full voltage drop on the desired cell makes the magnitude difference between its ON and OFF states much larger than any error introduced by sharing ‘*R*_*r*_’ or ‘*R*_*c*_’ over a segment. While both of ‘*R*_*r*_’ and ‘*R*_*c*_’ drain parasitic sneak-current, the current leak through only one of them affects the correctness of the readout operation. When the read circuit is connected to node ‘*n*_1_’, as shown in Fig. 4b, the sense current (*I*_*sense*_) is defined as,


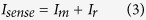


where ‘*I*_*m*_’ is the desired current and ‘*I*_*r*_’ is the row sneak current component. Sensing from node ‘*n*_2_’ swaps the locations and the role of ‘*R*_*r*_’ and ‘*R*_*c*_’ in the circuit, as shown in Fig. 5a. The sense current is shifted from its desired value by the sneak-current of the row or the column. However this shift is constant within a given row or column, based on the connection orientation.

## Initial Bits Readout

Each bit generally has two unknowns: ‘*R*_*m*_’ and ‘*R*_*r*_’ (or ‘*R*_*c*_’). Without adopting sneak-paths correlation and locality, multiple access stages are needed to estimate the bit value. However, a faster readout can be achieved by categorizing the bits into two types: the “initial bits,” which are the first bits accessed in a given column, and “regular bits,” which are any other bits in the array. To estimate the value of the “initial bit,” two unknowns need to be solved, namely the desired resistance (*R*_*m*_) and the row sneak resistance (*R*_*r*_). However, the remaining bits in the row share the same ‘*R*_*r*_’ value, and ‘*I*_*r*_’ is treated as the significant sneak-path component for a given row. Any of the readout techniques presented in the literature can be used to estimate the “initial bit”^21^. These “initial bits” readout dictates the threshold used for the remaining bits in that row. Figure 5a shows the readout sequence for the array when“initial bits” strategy is adopted. Therefore, the first (initial bit) could be any bit in the array that requires ‘*n*’ stages of reading. The rest of the bits in the same row are then accessed in sequence, only one time for each. Reading from the next row requires a new “initial bit”, which in this case is the first bit in the row, as shown in Fig. 5a. The same sequence is followed until the fetched data block for the cache is completed, i.e., each row contains one “initial bit”, and the rest of the bits are accessed in a single stage fashion. For a contiguous block of data readout using the “initial bits” technique, the proposed readout procedure is given as follows:

**Case 1:** The first accessed bit in the row ‘*i*’ (the initial bits),

- Use a multi stage readout technique to estimate the desired cell current 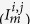 and the row sneak-current component 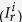.

**Case 2:** Accessing the rest of the bits in the same row,

- Access the desired cell for a single time to estimate its value, where ‘
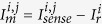
’.

where ‘*i*’ and ‘*j*’ are the desired row and column respectively. It should be noted that in the case of sensing from ‘*n*_1_’ data is accessed in a column-wise rather than row-wise scheme.

The readout circuitry for the “initial bit” is made of two parts: a virtual-ground ADC for the current sensing, and a digital processing circuitry for calculating the “initial bit” parameters and do the threshold comparisons. Typically, a single readout circuitry is needed per memory array. This does not impact the whole memory density as presented in previous works^3^.

## Predefined Dummy Bits Readout

A more time efficient way to estimate the adaptive threshold is to add “dummy bits” with predefined value to the array. The general concept of adding predefined bits to an array for sneak-paths estimation is presented in^31^. In our case, for a “dummy bit” the value of ‘*R*_*m*_’ is known in advance, and a single readout is needed to estimate the value of ‘*R*_*r*_’. This estimated ‘*R*_*r*_’ value is reused with the other bits in the same row. A single readout is required in this case to estimate the remaining unknown (*R*_*m*_). This value is used for the rest of the bits in the same row. The “dummy bit” can be organized in several ways, given that each row contains a single bit. Figure 5b shows a possible organization of dummy bits that is suitable for a row-wise readout analogy. For a contiguous block of data readout using the “dummy bits” technique, the proposed readout procedure is given as follows:

**Case 1:** Accessing the “dummy bit” of row ‘*i*’,

- Estimate the sneak-path row component using a single array access, where ‘
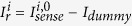
’.

The current ‘
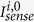
’ can be used without any processing, since the values of ‘*I*_*dummy*_’ is a DC shift that can be compensated in comparison process.

**Case 2:** Accessing the rest of the bits in the same row,

- Access the desired cell for a single time to estimate its value, where ‘
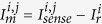
’.

where ‘*i*’ and ‘*j*’ are the desired row and column respectively. The dummy current ‘*I*_*dummy*_’ is known in the design time, where it can be ‘*I*_*on*_’ or ‘*I*_*off*_’ depending on which value is used to be stored in the dummy cells. Moreover, a dummy cell could be just a reference static resistor rather than a memristor, since there is no need to write it after the array fabrication.

**Figure 6:**
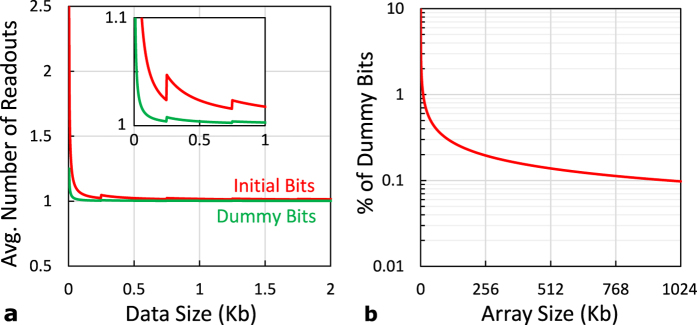
(**a**) The average number of readouts versus the fetched data size for the adaptive threshold techniques, where the first accessed bit is the middle of a row. (**b**) The percentage of “dummy bits” versus the array size.

The “dummy bits” technique adds a small overhead to the readout process, as a “dummy bit” needs to be accessed a single time (in comparison to ‘n’ times for an “initial bit”). However, for practical size arrays of 256 k size or more, the average number of array accesses per bit that occurs when fetching a block of data from memory is almost one for both methods. Figure 6a shows the average number of readouts per memory bit, where the overhead is shared over “regular bits”, versus the fetched data size. It also illustrates how the average number of readouts converges to one very fast. The ripples in the curve occur because that start reading from a new row adds extra overhead of an “initial bit” or a “dummy bit”. It should be noted that the typical cache line is 0.5 kb (64 bytes), where multiple lines are fetched from memory in sequence based on the cache policy. This value is much larger in the case of RAM fetching from HDD. While the “dummy bits” technique exhibits a better behavior, it comes at a small cost to the effective area of the array, as “dummy bits” are not used to store real data. This negligible overhead is shown in Fig. 6b.

The readout circuitry for the “dummy bits” technique can be implemented in two ways. The first approach is to use an analog circuit for current sensing and a simple digital circuit for comparisons and estimation, as discussed in the “initial bits” readout. Typically, most of the readout circuit area in this methodology is consumed by the conversion of the data from one domain to the other using ADCs^3^. A more area efficient implementation is to adopt a totally analog compensated readout circuit, as presented in previous work^23^. In this approach, the current of a “dummy cell” is sampled on a first capacitor, and the sensed current from each desired cell is sampled on a second one in sequence. Comparison between the two capacitor voltages leads to estimating the stored data in the desired memory cells^23^.

## Variability

Variability is a challenge that faces resistive-memory readout techniques. In general, two types of variability issues face memristor-based memory. The first is fabrication variability, in which fabricated cells have a distribution of ON and OFF values, rather than two unique states. The other source of variability is operation variability, in which the device parameters change stochastically or with aging. In the proposed readout, a memristor device variability can affect three types of cells. The first type is the “normal bits”, where any change in the cell properties can ruin its readout alone and impact the sneak-paths estimation. This is because the presented sneak-paths estimation techniques do not assume any properties of the “normal” cells, instead a group of parallel resistances whose effect as sneak-paths is probed for each new row readout. However, variability in the “normal bits” has a secondary impact on the read margins of the system, since it widens the distributions representing the possible ON and OFF values. The change in the read margins is defined as,


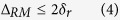


where *δ*_*r*_ is the maximum absolute shift in a cell value due to variability.

The second type of bit, which are “initial bits” does not suffer from any variability issues, because multi-stage readout techniques used to access such bits typically do not assume any properties of the probed cell. Such methods read and write the cell under probe multiple times during each readout to define a local threshold for the cell. Therefore, adopting multistage readout for accessing the “initial bits” makes the system less vulnerable to variability yet yields a very high total throughput. The last type of bits are “dummy bits”. Variability can impact the sneak-paths estimation since the dummy bit is used as a reference for the other cells in the row. Typically, variability in a “dummy bit” results in a threshold shift, which results in a decrease in read margins defined as


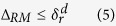


where 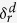 is the maximum absolute shift in a “dummy cell value” due to variability. The variability effect caused by a “dummy cell” can be reduced by storing the less variable memory stage (ON or OFF) in a dummy bit, or by using static resistance as a dummy cell rather than a memristor. Moreover, in the case of devices with high OFF resistance, storing “Zeros” in the dummy cell makes the probed current from its location much closer to the sneak-current rather than the cell current.

## Results and Discussions

In order to evaluate the validity and efficiency of crossbar readout techniques, an accurate simulation platform that includes different crossbar non-idealities is needed. To achieve this goal, we employ a Python script that creates SPICE netlists for realistic size arrays and sweeps different parameters and data patterns by calling HSPICE or Cadence APS iteratively. The test array can be filled with any predefined, random, or realistic workload as NIST RAM images^28^. Finally, the Python script braces the SPICE output files to collect the data of interest and tabulate it. We used a crossbar parasitic resistance value of 5 Ω per cell^3^ and included the effect of the switching row and column circuitry in all of the simulations in this work. For the memristor device, we adopted a bipolar device model proposed for memory operations^5^. Finally, it should be noted that resistive RAMs are built in the same hierarchy and structure of DRAMs, where subarrays of size up to 256 kb are used to reduce the capacitive loading of the metal lines^32^. Hence, we use an array size up to 256 kb for simulations and comparisons with this work.

### Error Free Readout

**Figure 7:**
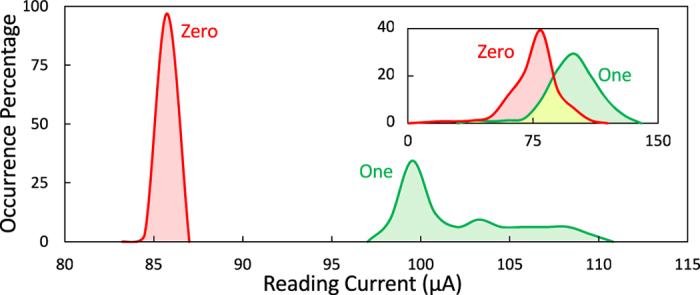
Histogram of the readout current for reading from a single row using the proposed readout technique. Inset: The original histogram of the readout as achieved without applying the adaptive threshold readout.

To verify the proposed concept, we simulated the readout operation at different locations of a 256 kb array of various NIST RAM images. In the first case, the readout locations are distributed over the array while in the second case all the readouts are made for cells in the same column. Figure 7 shows the histogram of the sensed read current for the two cases. The results indicate that the distributions of reading “One” and reading “Zero” are highly overlapped, and that it is not possible to define a threshold to distinguish between the two binary cases, as shown in Fig. 7 (inset). However, for a given row or column, reading from different locations reveals a clear separation between the distribution of ones and zeros, as shown in Fig. 7. This verifies our proposed readout scheme, in which an adaptive threshold is defined for each column (or row) as discussed earlier. The simulation results show that a simple comparator is required to differentiate between “One” and “Zero” states.

### Crossbar Power Consumption

**Figure 8:**
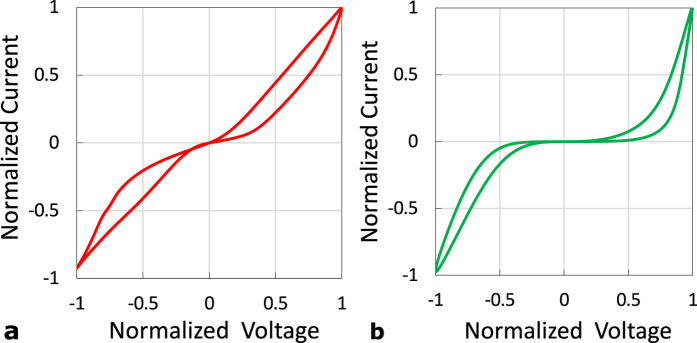
Measured ‘*i*-*v*’ hysteresis of two of our fabricated devices showing (**a**) low and (**b**) high saturation nonlinearity.

**Figure 9:**
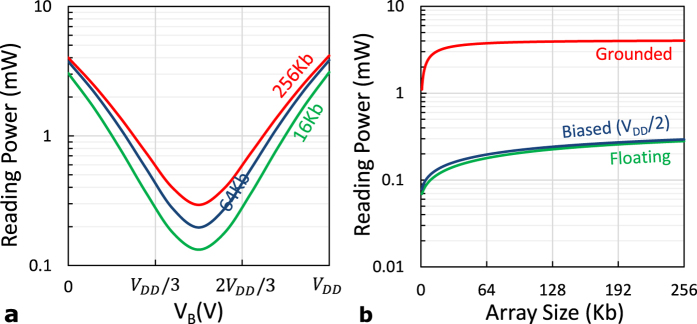
Reading power consumed by a biased-terminals crossbar filled with checkered data pattern versus (**a**) the bias voltage and (**b**) the array size.

Undesirable sneak-paths power consumption is not avoidable in high-density gateless arrays. However, it can be reduced by utilizing devices with nonlinear saturation behavior. Figure 8 shows the ‘*i*-*v*’ hysteresis of two of our fabricated devices. The second device shows higher saturation nonlinearity than the first one. Reducing the voltage applied to such devices by fifty percent can increase its saturation resistance up to two orders of magnitude^27^. This is a very attractive property since a sneak path is made of a series of memristor devices, where a sub-voltage is dropped on each of them. In the “connected terminals” structure, the device nonlinearity can be enforced by biasing the unused terminals to sub-read voltage. In such case, the very small ‘*R*_*a*_’ is shorted out, and the nonlinearity of the other terminals efficiently utilized. Figure 9a shows that the optimal selection is made by biasing the unused terminals voltage to be *V*_*B*_ = *V*_*DD*_/2. The power consumption of this method is almost the same as the baseline “floating terminals”, as shown in Fig. 9b. The figure also shows the great power-saving of the “connected terminals” while comparing it with the power hungry “grounded terminals” technique. It should be noted that power consumption saturates for larger array sizes because of the crossbar metal lines^3^.

### Figure-of-Merit

In general, the presented technique offers a sneak-paths immune readout that is more power efficient and faster than the state-of-the-art crossbar accessing techniques that are presented in the literature. Table 1 shows a detailed comparison of the various gateless techniques that can provide an error-free readout. The different methods are compared based on a figure-of-merit (FoM), which is defined as,


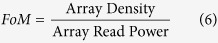


where the proposed technique shows the best FoM.

## Conclusion

Taking advantage of memory locality and the sneak-paths correlation yields a fast and power efficient readout technique. Unlike other techniques, the proposed method achieves the theoretical limit of a single memory access per pixel for an array readout at a fraction of the power of state-of-the-art readout techniques. The presented adaptive-threshold readout is 7 to 24 times better than the other gateless techniques presented in the literature, based on the density-power figure-of-merit. In addition, the new sneak-paths immune technique requires minimal hardware to distinguish between the memory data values.

## Additional Information

**How to cite this article**: Zidan, M. A. *et al.* Single-Readout High-Density Memristor Crossbar. *Sci. Rep.* **6**, 18863; doi: 10.1038/srep18863 (2016).

## Figures and Tables

**Table 1:** Comparison between state-of-the-art gateless readout techniques for a subarray of size 256 kb.

	Error Free Readout	# of Reads	# of Writes	Locality Needed	Readout Circuit^*^	Read Power [mW]	FoM [*Tbit*/*cm*^2^*W*]
Multi-Stage^5^	Yes	3	3	No	ADC + Comp	7	91
Multi-Port^3^	Yes	3	0	No	ADC + Comp	2.1	304
Grounded Rows & Cols^26^	No	1	0	No	VG + Comp	4	160
**This Work (initial bits)**	Yes	1.05^**^	< 0.01	Yes	ADC + Comp	**0.293**	**2184**
**This Work (dummy bits)**	Yes	1.01^**^	0	Yes	VG + Comp	**0.291**	**2195**

^*^ADC: Analog-to-Digital Converter, Comp: Comparator, and VG: Virtual-Ground.

^**^The number of reads is calculated for the case of 16 bytes being fetched from the array in sequence.
